# Catheter-Directed Thrombectomy: An Alternative in Massive Pulmonary Embolism

**DOI:** 10.1155/2022/3562017

**Published:** 2022-04-11

**Authors:** Andres Cordova Sanchez, Mostafa Vasigh, Oluwateniola Olatunde, Debanik Chaudhuri

**Affiliations:** ^1^Department of Medicine, SUNY Upstate Medical University, Syracuse, NY 13210, USA; ^2^Division of Cardiology, SUNY Upstate Medical University, Syracuse, NY 13210, USA

## Abstract

Massive pulmonary embolism (PE) is a life-threatening condition. The mainstay treatment is thrombolysis. Catheter-directed thrombectomy involves a group of new techniques that appear to have relatively low complications and mortality. These techniques have so far been studied mostly in submassive PE. We present a patient with massive PE that was successfully treated with catheter-directed thrombectomy.

## 1. Introduction

Massive PE is defined as acute PE with systolic blood pressure<90 mmHg for >15 minutes, ≥40 mmHg drop from baseline, or requiring hemodynamic support [1]. It has a mortality risk of 43% to 62% compared to around 15% for nonmassive PE [2].

Its management consists of early anticoagulation plus thrombolysis or thrombectomy. The mainstay treatment is thrombolysis, although it has several contraindications.

Catheter-directed therapies can be divided into directed thrombolysis and embolectomy. Catheter-directed thrombectomy is a reasonable option when there are contraindications for thrombolysis. The available systems are Flowtriever, Penumbra Indigo, and AngioVac. These thrombectomy systems are still being studied. Most studies so far have focused on submassive PE. To our knowledge, there are no studies focusing on massive PE.

We present a patient with massive PE and cardiac arrest that underwent a successful percutaneous embolectomy with a Penumbra Indigo Cat 12 device.

## 2. Case

**Figure 1:**
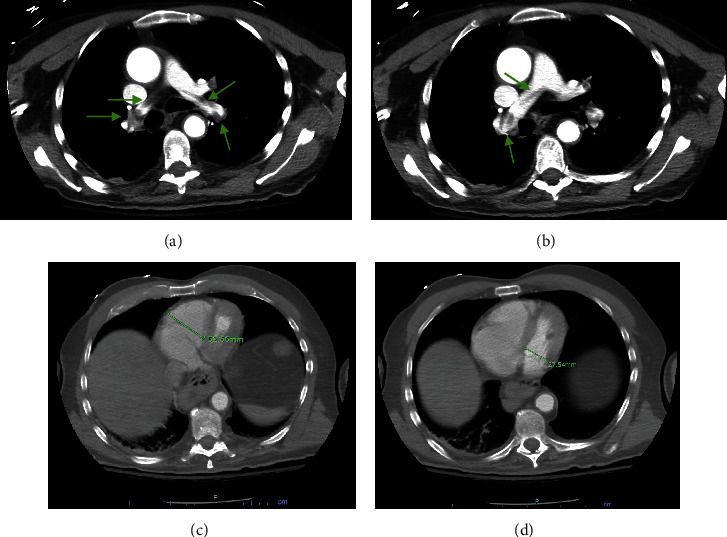
(a, b) Pulmonary embolism on the left and right pulmonary arteries. (c, d) RV and LV measurement demonstrating an RV/LV diameter of 1.9, septal bowing to the left further supports right ventricular strain.

A 73-year-old man with past medical history significant for upper gastrointestinal bleed 1 month ago and ischemic stroke with residual aphasia and right-sided hemiplegia 3 months before admission, respectively. He was transferred from an outside hospital for sudden acute hypoxic respiratory failure requiring 5 liters of oxygen via nasal cannula and cardiogenic shock on epinephrine and norepinephrine support. A computed tomography angiography of the chest showed a saddle PE with large thrombus burden in both right and left pulmonary arteries, leftward septal deviation, right ventricle (RV) 52.56 mm, left ventricle (LV) 27.55 mm, and RV/LV ratio of 1.9 (Figure 1). He was started on a heparin drip. En route to our hospital, he became pulseless and unresponsive. Cardiopulmonary resuscitation was started; return of spontaneous circulation was obtained after 2 rounds of epinephrine. Postarrest, he regained baseline mental status.

He was not a candidate for TPA given recent cerebral infarct and gastrointestinal bleeding; thus, he was planned for thrombectomy.

On arrival, he was still on obstructive shock, requiring two pressor support. He was requiring 4 liters of oxygen via nasal cannula to saturate above 90%. Soon after arrival, percutaneous thrombus aspiration with a Penumbra Indigo embolectomy CAT 12 device was done. Pulmonary angiogram after the procedure showed near-complete resolution of thrombus burden. A small residual thrombus remained despite multiple passes with the device. No complications occurred during the procedure. Postthrombectomy systolic pulmonary artery pressure dropped to 43 mmHg from 47 mmHg prethrombectomy. The patient was successfully weaned off pressors and oxygen shortly after. Doppler ultrasound of the lower extremities showed a right lower extremity deep vein thrombosis. An echocardiogram done approximately 10 hours after thrombectomy was a normal study with an ejection fraction of 70%, normal left and right ventricular function, and estimated pulmonary arterial pressure of 23 mmHg.

He remained hemodynamically stable during the rest of his hospitalization, was eventually transitioned to apixaban, and discharged on the 5th day of hospitalization.

## 3. Discussion

Massive PE is a life-threatening condition that requires time-sensitive management and carries high mortality risk. When thrombolysis has failed or in cases with absolute contraindications, catheter or surgical embolectomy is to be considered [1].

The Penumbra Indigo system uses a catheter connected to a suction device. To date, there is only one prospective study investigating this device, the EXTRACT-PE trial. However, it was done almost exclusively in submassive PE and had only one patient enrolled with massive PE. They found a mean reduction of RV/LV ratio of 0.43, all-cause mortality at 30 days of 2.5%, and a median introduction to removal time of 37 minutes, and major bleeding was seen in approximately 1.7% [3] compared to 10% to 11.5% with thrombolysis [4, 5]. Of the 119 patients included in the study, there was one patient death and one device-related distal artery perforation [3]. The FLARE trial looked at the use of the Flowtriever system in patients with submassive PE; it demonstrated a reduction of the RV/LV ratio of 0.38, 3.8% of major complications, and an average procedural time of 94 minutes [6].

Surgical embolectomy is an invasive procedure that classically requires median sternotomy, cardiopulmonary bypass, and pulmonary trunk excision. A minimally invasive procedure also exists [7]. Operative time appears to be similar in both classical and minimally invasive approaches at 160 minutes to 251 minutes [8]. Mortality has been reported at 6% to 23.7% for massive and 3.6% to 9.1%for submassive [9–11].

Given these results, it appears that catheter embolectomy is a safe and fast approach in patients with submassive PE. However, to our knowledge, no studies have focused on massive PE.

Catheter-directed thrombolysis uses low dose thrombolytic infusion directly into the PE and is an option for patients with pulmonary embolism that have relative contraindication to systemic thrombolysis [1]. Preliminary results from the PERFECT registry showed the use of catheter-directed thrombolysis in eight patients with absolute contraindications for systemic thrombolytics without any major bleeding events [12]. We considered that, in our case, the risk of a major bleeding event was too high on the account of recent gastrointestinal bleeding and cerebrovascular event. Thus, only catheter embolectomy without the use of any thrombolytics was done.

**Figure 2:**
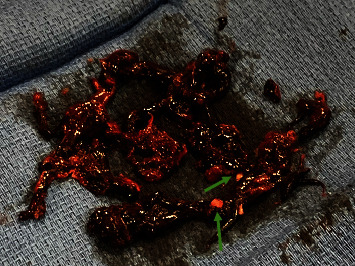
Thrombus removed during thrombectomy. White appearing thrombus (arrows) suggesting chronically organized thrombus.

Our patient presented with massive PE and high risk of bleeding. He was treated with catheter embolectomy using a Penumbra Cat 12 device with excellent results. A small residual thrombus remained despite several passes with the device; this could have been a chronically organized thrombus attached to the vessel wall, which was supported by the presence of white, chronically appearing aspirate (Figure 2).

## 4. Conclusions

Catheter-directed embolectomy could be considered a safe and fast alternative for the management of massive PE in patients that have failed or are not candidates for thrombolysis. These findings will need to be confirmed in randomized controlled trials.

The presence of chronically organized thrombus in patients with acute massive PE could complicate the use of catheter-directed thrombectomy.
